# Anti-Inflammatory and Analgesic Properties of *Polygonum minus*: Mechanistic Insights and Therapeutic Potential

**DOI:** 10.3390/biomedicines14092070

**Published:** 2026-09-15

**Authors:** Mohd Amir Kamaruzzaman, Ruzaidi Ruslan, Isma Liza Mohd Isa, Nur Aqilah Kamaruddin, Elvy Suhana Mohd Ramli

**Affiliations:** 1 Departments of Anatomy, Faculty of Medicine, Universiti Kebangsaan Malaysia, Jalan Yaacob Latif, Bandar Tun Razak, Cheras, Kuala Lumpur 56000, Malaysia; mohdamir@ukm.edu.my (M.A.K.); ruzaidiruslan@gmail.com (R.R.); ismaliza.mohdisa@ukm.edu.my (I.L.M.I.); nur.aqilah@ukm.edu.my (N.A.K.); 2 CÚRAM, The Research Ireland Centre for Medical Devices and Rinn Medical Devices, University of Galway, H91 W2TY Galway, Ireland

**Keywords:** inflammation, pain, *Polygonum minus* (PM), anti-inflammatory, antioxidant

## Abstract

Inflammation is a complex protective biological response triggered by harmful stimuli such as pathogens, chemical agents, or physical injury, and is closely associated with the development of pain and various chronic diseases. It involves a coordinated cascade of cellular and molecular events, including activation of pattern recognition receptors, release of pro-inflammatory cytokines, production of reactive oxygen species, and recruitment of immune cells. Acute inflammation is essential for host defense and tissue repair. However, persistent or dysregulated inflammation may lead to chronic inflammation, oxidative stress, and tissue damage. Inflammatory pain, a subtype of nociceptive pain, arises from the sensitization of peripheral and central nociceptive pathways by inflammatory mediators such as cytokines, prostaglandins, and nitric oxide. Herbal products have been traditionally used to manage inflammatory diseases due to their bioactive compounds, which modulate inflammatory pathways and alleviate symptoms, and gaining increasing attention due to their multi-targeted mechanisms and lower adverse effects. *Polygonum minus* (PM), a medicinal herb widely used in Southeast Asia, is rich in bioactive compounds including flavonoids, phenolics, polygodial and essential oils. Phytochemical studies have identified compounds such as qsiuercetin and myricetin, which exhibit strong antioxidant and anti-inflammatory properties. Experimental evidence suggested that *PM* exerts its anti-inflammatory effects through the inhibition of key inflammatory pathways, including cyclooxygenase (COX) and lipoxygenase (5-LOX), suppression of pro-inflammatory cytokines, and modulation of nuclear factor-kappa B (NF-κB) signaling. Additionally, its antioxidant activity contributes to the reduction in oxidative stress, thereby limiting inflammation-induced tissue damage. This review highlights the mechanisms the potential therapeutic role of PM in modulating underlying inflammation and inflammatory pain. The evidence suggested that PM may serve as a potential promising natural agent for the management of inflammation and pain. Further studies are warranted to elucidate its molecular mechanisms and to validate its efficacy and safety in clinical settings.

## 1. Introduction

### 1.1. Background on Pain and Inflammation

Inflammation is a protective biological response that occurs when tissue homeostasis is disrupted by biological, chemical, or physical stimuli [1]. It can be broadly classified into acute and chronic inflammation, depending on the nature of the stimulus and the effectiveness of the body’s response to tissue injury. The inflammatory response is influenced by the nature of the initial stimulus and its location within the body. Although the triggers and sites of inflammation may vary, the underlying mechanisms are generally similar. The inflammatory process typically involves several sequential events: (1) recognition of harmful stimuli by cell-surface receptors, (2) activation of intracellular signaling pathways, (3) secretion of inflammatory mediators, and (4) recruitment of inflammatory cells to the affected tissue [2,3].

The inflammatory response involves the recruitment and activation of various inflammatory cells at the site of tissue injury. When tissue damage occurs, epithelial and endothelial cells release mediators that initiate the inflammatory cascade, including chemokines and growth factors that attract immune cells such as neutrophils and monocytes. Neutrophils are among the earliest cells recruited to the site of inflammation and play a critical role in the initial immune response. They participate in pathogen clearance, release inflammatory mediators, and contribute to antigen presentation processes, which are essential for activating T lymphocytes and facilitating the recruitment of additional immune cells, including monocytes and dendritic cells.

Acute inflammation serves as a protective and restorative mechanism activated by injury or infection [4]. It develops rapidly, usually within minutes, may persist for several hours or days, and is characterized by an increase in vascular permeability, exudation of plasma proteins and fluids, and the migration of leukocytes—particularly neutrophils—to the site of injury. It causes the release of pro-inflammatory mediators, including bradykinin, prostaglandins, and leukotrienes. The inflammatory response resolves and tissue homeostasis will be restored when the immune system successfully neutralizes and eliminates the causative agent. However, if the offending stimulus persists or the immune response is inadequate, the inflammatory process may progress to chronic inflammation [5].

Chronic inflammation is a prolonged and dysregulated inflammatory response that occurs when the inciting stimulus is not effectively eliminated or when normal resolution mechanisms fail. Chronic inflammation is typically associated with the infiltration of lymphocytes and macrophages, vascular proliferation, fibrosis, and progressive tissue damage [6]. These cells continuously release pro-inflammatory cytokines such as tumor necrosis factor-α (TNF-α), interleukin-1 (IL-1), and interleukin-6 (IL-6), thereby sustaining immune cell recruitment and establishing a self-perpetuating cycle of inflammation. The process is further amplified by the activation of intracellular signaling pathways, including nuclear factor-kappa B (NF-κB), mitogen-activated protein kinase (MAPK), and Janus kinase–signal transducer and activator of transcription (JAK–STAT) pathways, which promote the expression of inflammatory genes and mediators such as cyclooxygenase-2 (COX-2) and inducible nitric oxide synthase (iNOS). In addition, activated inflammatory cells generate reactive oxygen species (ROS), leading to oxidative stress that damages cellular components, including lipids, proteins, and DNA, while further enhancing inflammatory signaling. Failure of anti-inflammatory and resolution pathways further contributes to the persistence of inflammation, leading to progressive tissue damage and is implicated in the pathogenesis of various chronic diseases, including autoimmune disorders, cardiovascular diseases, metabolic syndrome, and cancer [6].

Inflammation is characterized by five cardinal signs: redness (rubor), swelling (tumor), heat (calor), pain (dolor), and loss of function (functio laesa) [7,8]. Inflammation was regarded primarily as an immediate immune response to tissue injury or infection by pathogens such as bacteria and viruses. However, advances in molecular biology, genetics, and pharmacology have revealed that inflammation is a highly complex and regulated process involving multiple cellular and molecular pathways. Contemporary research increasingly focuses on identifying genetic predispositions, cellular signaling mechanisms, and novel therapeutic targets for the prevention and treatment of inflammatory diseases [3,8].

Following neutrophil infiltration, monocytes, lymphocytes, and mast cells are recruited to the inflamed tissue, expressing pattern recognition receptors (PRRs), which are also present in non-immune cells such as epithelial cells and fibroblasts. These receptors detect pathogen-associated molecular patterns (PAMPs) and damage-associated molecular patterns (DAMPs) during infection or tissue injury, thereby initiating signaling pathways that promote leukocyte recruitment and activation at sites of inflammation. Once in the tissue, monocytes differentiate into macrophages, which are the key regulators of the inflammatory response. Activated macrophages release a wide range of pro-inflammatory mediators, including cytokines such as tumor necrosis factor-α (TNF-α), interleukin-1 (IL-1), interleukin-6 (IL-6), interleukin-8 (IL-8), and interleukin-12 (IL-12). This will further activate intracellular signaling cascades through receptors such as Toll-like receptors (TLRs), IL-1 receptors, IL-6 receptors, and TNF receptors, leading to the production of inflammatory mediators, such as chemokines, leukotrienes, prostaglandins, and complement proteins, all of which interact to amplify and coordinate the inflammatory response [5].

Activated inflammatory cells, particularly macrophages, release a range of mediators, including cytokines, acute-phase proteins, and oxidative enzymes. Elevated levels of IL-1β, IL-6, TNF-α, C-reactive protein (CRP), and haptoglobin are characteristic of inflammatory responses. Additionally, enzymes associated with oxidative stress, such as superoxide dismutase (SOD), glutathione peroxidase (GPx), NADPH oxidase (NOX), inducible nitric oxide synthase (iNOS), and cyclooxygenase-2 (COX-2), are upregulated. These processes generate oxidative stress markers, including malondialdehyde (MDA), 8-hydroxy-2′-deoxyguanosine (8-OHdG), and isoprostanes, which serve as indicators of inflammation and cellular damage [9].

While the inflammatory response is essential for protecting the host and promoting tissue repair, excessive or uncontrolled production of inflammatory mediators can be harmful. When cytokines are produced in appropriate amounts, they help eliminate pathogens and restore tissue homeostasis. However, overproduction of pro-inflammatory cytokines may lead to tissue damage and organ dysfunction [10]. For instance, excessive release of IL-1β and TNF-α can trigger a systemic inflammatory response, which is a hallmark of septic shock and may ultimately result in multi-organ failure [11].

The inflammatory process, which serves as a key defense mechanism against pathogens, is closely associated with the production of reactive oxygen species (ROS). During inflammation, activated immune and inflammatory cells generate elevated levels of ROS as part of the host defense system to eliminate invading microorganisms. While moderate ROS production is beneficial for microbial killing and immune signaling, excessive or prolonged ROS generation can lead to oxidative stress, resulting in cellular and tissue damage and contributing to the development of chronic inflammation. Consequently, oxidative stress and inflammation are closely interconnected processes that can amplify each other. Persistent oxidative or inflammatory stress may ultimately lead to cellular dysfunction and death through mechanisms such as necrosis, apoptosis, cellular senescence, or adaptive survival responses, such as autophagy, which involves the degradation of damaged cellular components [5].

Natural products derived from medicinal plants have gained increasing attention as potential sources of anti-inflammatory and analgesic agents. Many plant-based compounds possess bioactive constituents such as flavonoids, phenolic acids, and terpenoids, which exhibit antioxidant, anti-inflammatory, and analgesic properties. These compounds may exert their therapeutic effects by inhibiting the production of pro-inflammatory cytokines, suppressing oxidative stress, and modulating key signaling pathways involved in inflammation and pain [12].

### 1.2. Polygonum minus (Kesum)

Among traditionally used medicinal plants, *Polygonum minus* has attracted considerable research interest because of its reported anti-inflammatory and analgesic properties. *Polygonum minus* Huds. (synonym: *Persicaria minor* (Huds.) Opiz), commonly known as “kesum” in Malaysia, is a widely used culinary herb and traditional medicinal plant in Southeast Asia. It belongs to the family Polygonaceae, is a small, aromatic, herbaceous annual perennial plant native to Southeast Asia, particularly Malaysia, Indonesia, Thailand, and Vietnam. It is also available in temperate regions of Europe. Universally known as Daun Kesum in Malaysia, Indonesia and Brunei, Phak Phai in Thailand and Rhu and Rau ram In Vietnam, as well as *Pygmy smartweed* in English. It thrives in damp, waterlogged habitats such as riverbanks, marshlands, and ditch margins. It is a low-growing, slender herb reaching 10–40 cm in height. The stems are decumbent-ascending, cylindrical, highly branched, and root easily at the lower nodes. It has simple, alternate leaves that are lanceolate to linear-lanceolate, measuring 2–7 cm in length and 0.5–1.5 cm in width. The leaves possess short petioles, smooth margins, acute tips, and a distinct dark green, glossy lamina. Crushed leaves release a pungent, sharp, citrusy aroma due to high concentrations of volatile aldehyde constituents (e.g., decanal, dodecanal). The ochrea, a tubular, membranous stipular sheath fringed with fine cilia encasing the base of each petiole node, is the characteristic feature of this herbal plant. The flowers are minute, actinomorphic, with a 5-lobed tepal structure varying in color from pale pink to white [13,14,15].

*P. minus* is an essential culinary herb valued for its flavor-enhancing and preservation properties which is widely used in Southeast Asian cuisine, such as *Laksa*, *Asam Pedas*, *Nasi Ulam*, and *Kerabu* (traditional salad). It has long been utilized as a traditional therapeutic agent due to its ability to alleviate gastrointestinal spasms, inflammatory skin disorders, and musculoskeletal pain. In addition, the essential oil extracted from the plant is traditionally used for conditions such as dandruff [13,14,15].

### 1.3. Scope and Objectives of the Review

This review comprehensively covers scientific literature on *P. minus* published across peer-reviewed databases (including PubMed, Scopus, Web of Science, and Google Scholar). Despite the extensive traditional use of *Polygonum minus* (syn. *Persicaria minor*) across Southeast Asia as a folk remedy for painful and inflammatory conditions, a comprehensive synthesis unifying its phytochemical constituents with modern molecular pharmacology remains lacking. Previous literature has largely focused on its general antioxidant capacity or culinary application, leaving a critical gap in understanding how its specific bioactives modulate central and peripheral nociceptive pathways as well as inflammatory cascades.

In this context, *Polygonum minus* (PM) has demonstrated promising anti-inflammatory activity in carrageenan-induced edema models. The observed effects are likely attributed to its rich phytochemical composition, particularly flavonoids such as quercetin and myricetin, which are known to inhibit the synthesis of inflammatory mediators. Experimental findings suggest that PM extract significantly reduces paw edema, especially during the late phase of inflammation, indicating its potential to suppress prostaglandin-mediated pathways. Furthermore, its dual inhibitory action on cyclooxygenase (COX) and 5-lipoxygenase (5-LOX) pathways may contribute to the reduction in both prostaglandin and leukotriene production, thereby limiting the accumulation of inflammatory mediators and reducing tissue damage [16]. An anti-inflammatory effect was also observed in vivo in another closely related plant species, Polygonum hydropiper [13]. These findings support the potential therapeutic role of PM as a natural anti-inflammatory agent.

## 2. Molecular Basis of Inflammation and Pain

Pain is defined by the International Association for the Study of Pain (IASP) as an unpleasant sensory and emotional experience that is associated with actual or potential tissue damage. It serves as an important protective mechanism, alerting the body to harmful stimuli and prompting appropriate responses to prevent further tissue injury [17].

Pain can be broadly classified into nociceptive pain and neuropathic pain. Nociceptive pain arises from the activation of nociceptors, which are specialized sensory receptors located in the peripheral somatosensory nervous system. These receptors respond to potentially harmful stimuli, such as mechanical, thermal, or chemical damage to non-neural tissues. The resulting signals are transmitted to the central nervous system, leading to the perception of pain. Common examples of nociceptive pain include pain caused by skin incisions, inflammation, or superficial injuries [15].

In contrast, neuropathic pain results from damage or dysfunction within the nervous system itself, particularly involving the somatosensory pathways. This type of pain is often associated with conditions such as diabetic neuropathy or spinal cord injury, in which structural or functional abnormalities in neural tissues lead to persistent pain sensations.

Inflammatory pain is considered a subtype of nociceptive pain and occurs as a consequence of inflammatory processes within damaged tissues, with inflammation playing a central role in the initiation and maintenance of pain. It is mediated by the release of various inflammatory mediators. Following tissue injury, pro-inflammatory mediators such as bradykinin, prostaglandins, and leukotrienes are released, leading to the activation and sensitization of nociceptors, which are specialized receptors responsible for pain detection. These mediators lower the activation threshold of nociceptors, resulting in hyperalgesia and increased sensitivity to pain at the site of injury [18,19]. At the molecular level, these mediators stimulate key intracellular signaling pathways, particularly nuclear factor-kappa B (NF-κB) and mitogen-activated protein kinase (MAPK) pathways. Activation of NF-κB leads to the transcription of pro-inflammatory genes encoding cytokines, chemokines, cyclooxygenase-2 (COX-2), and inducible nitric oxide synthase (iNOS), thereby amplifying the inflammatory response and nociceptive signaling. Similarly, MAPK pathways, including extracellular signal-regulated kinase (ERK), c-Jun N-terminal kinase (JNK), and p38 MAPK, contribute to the regulation of inflammatory gene expression and neuronal sensitization [17].

The cyclooxygenase (COX) pathway, particularly COX-2, plays a crucial role in converting arachidonic acid into prostaglandins such as prostaglandin E2 (PGE2), which lowers the activation threshold of nociceptors and enhances pain transmission [20]. In addition, ROS generated during inflammation can further activate NF-κB and MAPK signaling, creating a positive feedback loop that sustains both inflammation and pain. Beyond peripheral sensitization, these molecular events also contribute to central sensitization within the spinal cord and brain, where increased excitability of nociceptive neurons leads to amplified and prolonged pain responses [15].

The perception of pain ultimately involves the integration of a series of sensory signals by the brain, allowing the body to recognize potentially harmful stimuli and generate appropriate protective responses. While acute inflammatory pain is protective and promotes tissue repair, persistent activation of these molecular pathways can result in chronic pain through continuous nociceptor sensitization and maladaptive neuronal plasticity [15]. Therefore, targeting key inflammatory pathways, including NF-κB, MAPK, and COX-2, represents an important therapeutic strategy for the management of inflammatory and chronic pain.

### Assessment of Pain in Experimental Animals

Preclinical analgesic activity is commonly evaluated using behavioral models that assess mechanical, thermal, and chemical nociceptive responses. Mechanical sensitivity is typically measured using von Frey-based assays, whereas hot-plate, tail-flick, and Hargreaves tests assess responses to noxious thermal stimuli. Chemical and inflammatory models, particularly formalin-induced nociception and carrageenan-induced inflammation, are useful for distinguishing peripheral and central analgesic mechanisms and for evaluating modulation of inflammatory mediators. These complementary models have been widely employed to characterize the antinociceptive and anti-inflammatory activities of natural products, including *Polygonum minus* extracts [21,22].

## 3. Phytochemical Profile and Bioactive Constituents of *Polygonum minus*

Phytochemical investigations of PM have identified several bioactive compounds, particularly flavonoids and essential oils. Reported flavonoids include myricetin and quercetin, as well as methylated flavanol and flavone derivatives such as 6,7-4′,5′-dimethylenedioxy-3,5,3′-trimethoxyflavone and 6,7-methylenedioxy-5,3′,4′,5′-tetramethoxyflavone [23,24]. These compounds are known for their antioxidant and anti-inflammatory properties.

The plant is also rich in essential oils, constituting approximately 72.54% of its volatile components. These oils are predominantly composed of aliphatic aldehydes (approximately 87%), with decanal and dodecanal being the major constituents. Other components include 1-decanol, 1-dodecanol, undecanal, tetradecanal, 1-undecanol, nonanal, 1-nonanol, and β-caryophyllene [25]. Advanced analyses using gas chromatography–mass spectrometry have identified up to 69 volatile compounds in the leaf essential oil [25]. Notable compounds such as geraniol and geranial (citral) contribute to the plant’s characteristic aroma and exhibit antimicrobial, antioxidant, immunostimulatory, and anticancer activities [26]. Additionally, the presence of oxalic acid in the leaves has been suggested to contribute to its digestive-enhancing effects [22].

The most distinctive and pharmacologically signature substance in *Polygonum minus* is polygodial, a drimane-type sesquiterpene dialdehyde. It is responsible for the sharp, peppery taste and hot sensation when raw leaves are consumed. It acts as a potent TRPV1 (Transient Receptor Potential Vanilloid 1) agonist, initially activating a transient pain/burning sensation followed by rapid receptor desensitization, leading to peripheral analgesia. Inhibits pro-inflammatory mediator release (TNF-alpha, PGE2) and exhibits strong antifungal activity against *Candida* species and foodborne pathogens. Polygodial also has the ability to inhibit pro-inflammatory mediator release (TNF-alpha, PGE2) and exhibits strong antifungal activity against *Candida* species and foodborne pathogens [13].

Although studies directly investigating the anti-inflammatory activity of PM are limited, evidence from related species provides valuable insights. For example, Polygonum hydropiper has demonstrated significant anti-inflammatory effects both in vitro and in vivo, including the inhibition of pro-inflammatory gene expression such as cyclooxygenase-2 (COX-2), tumor necrosis factor-alpha (TNF-α), nuclear factor kappa B (NF-κB), and prostaglandins [21]. Molecular systematic studies have shown strong genetic similarity between *P. hydropiper* and PM, with analyses using molecular markers demonstrating nearly 100% similarity across several accessions [27]. These findings suggest that PM may share similar anti-inflammatory mechanisms.

Mechanistically, PM extract is proposed to exert anti-inflammatory effects through the inhibition of both cyclooxygenase (COX) and 5-lipoxygenase (5-LOX) pathways that are involved in arachidonic acid metabolism. Unlike conventional non-steroidal anti-inflammatory drugs (NSAIDs), which primarily inhibit COX-mediated prostaglandin synthesis, PM may also suppress leukotriene formation via inhibition of the 5-LOX pathway. This dual inhibitory action may prevent the accumulation of key inflammatory mediators, thereby reducing tissue damage and enhancing its therapeutic potential [28,29].

**Figure 1:**
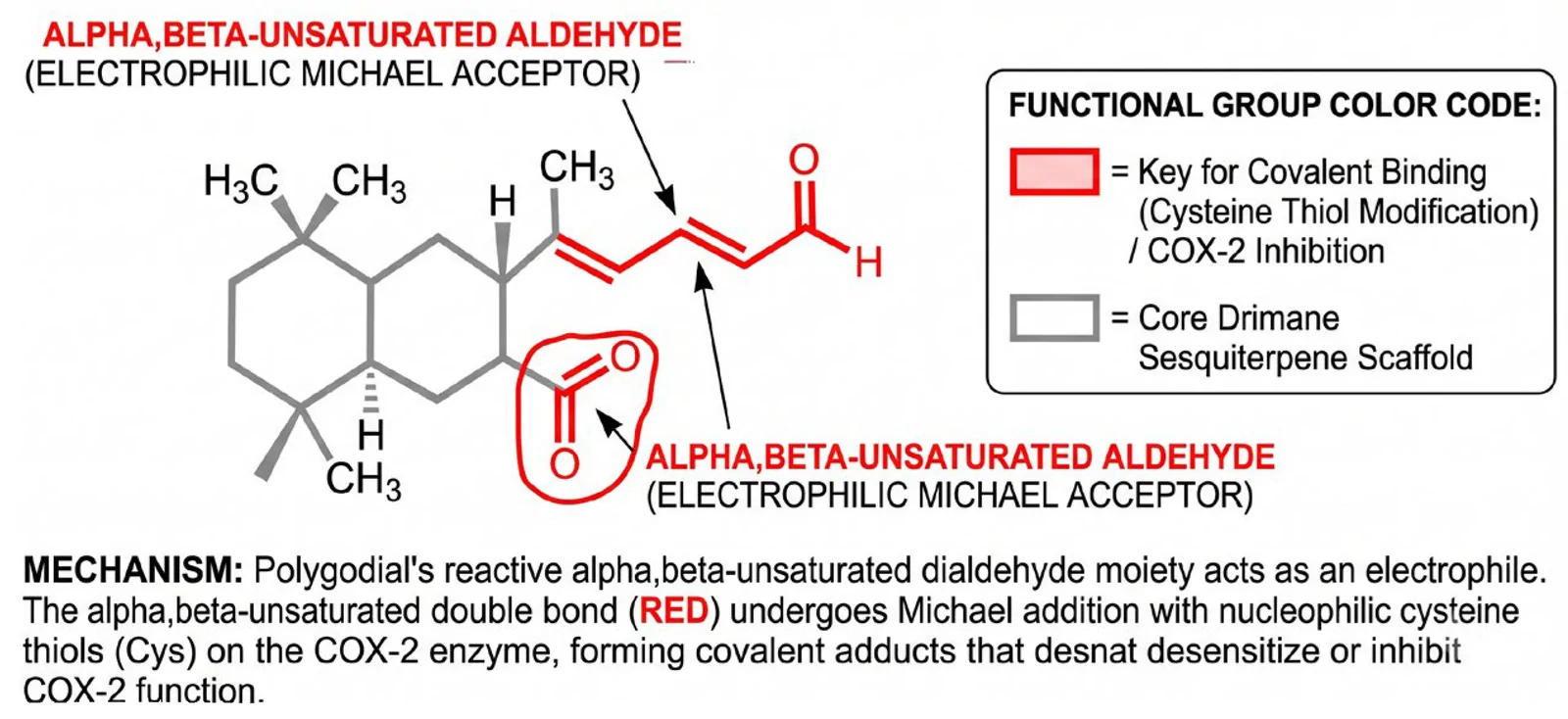
Chemical structure of polygodial [18].

Polygodial is a highly lipophilic and chemically reactive drimane sesquiterpene dialdehyde, and its bioavailability is expected to be strongly dependent on the route of administration. Although formal pharmacokinetic studies comparing the systemic bioavailability of polygodial following different routes of administration appear to be limited, pharmacological studies have demonstrated route-dependent activity. Polygodial produces antinociceptive effects following oral and intraperitoneal administration, with oral administration being approximately 2–8-fold less potent than intraperitoneal administration in the formalin test, suggesting differences in systemic exposure and/or disposition between these routes [30,31]. The structure is ilustrated in Figure 1.

### 3.1. Traditional Medicinal Uses of PM

Several bioactive constituents have been identified via phytochemical investigations of PM, particularly flavonoids and essential oils, which are believed to contribute to its pharmacological properties (Table 1). Among the flavonoids reported are myricetin [3,5,7-trihydroxy-2-(3,4,5-trihydroxyphenyl)-4-chromenone] and quercetin [2-(3,4-dihydroxyphenyl)-3,5,7-trihydroxy-4H-chromen-4-one]. Other compounds identified include a methylated flavanol, 6,7-4′,5′-dimethylenedioxy-3,5,3′-trimethoxyflavone, and a flavone derivative, 6,7-methylenedioxy-5,3′,4′,5′-tetramethoxyflavone [23,24]. These flavonoids are well known for their antioxidant and anti-inflammatory activities.

### 3.2. Pharmacological Activities of PM

#### 3.2.1. Anti-Inflammatory Activity

Inflammation plays a critical role in the pathogenesis of many chronic diseases, including arthritis, cardiovascular disease, and metabolic disorders. Several studies have demonstrated that extracts of PM possess significant anti-inflammatory activity both in vitro and in vivo, as presented in Table 2.

Flavonoids such as quercetin and myricetin, which are present in PM, are known to suppress inflammatory pathways by inhibiting the expression of pro-inflammatory mediators such as tumor necrosis factor-α (TNF-α), interleukin-1β (IL-1β), and cyclooxygenase-2 (COX-2) [14]. These compounds may also suppress the activation of nuclear factor-kappa B (NF-κB), a key transcription factor involved in inflammatory signaling [34].

Although studies directly investigating the anti-inflammatory activity of PM remain limited, research on closely related species provides valuable insights. For example, Polygonum hydropiper has demonstrated significant anti-inflammatory effects both in vitro and in vivo, including the inhibition of pro-inflammatory gene expression such as COX-2, TNF-α, NF-κB), and prostaglandins [13].

Molecular systematic studies have shown strong genetic similarity between *P. hydropiper* and PM, with analyses using molecular markers demonstrating nearly 100% similarity across several accessions [27]. A study done on the same carrageenan induced paw edema model, administrating Lepisanthes alata (LA) 250 mg/kg, a tropical plant that is rich in phytochemicals like polyphenols, with potent anti-inflammatory and antioxidant properties significantly decreased rat grimace score (RGS) as well as IL-1β, TNF-α, and histological inflammation with a positive effect on T-SOD levels [28]. These findings suggest that PM may share similar anti-inflammatory mechanisms that can act as a potential antioxidant (Table 2).

The aqueous extract of *P. minus* demonstrated a mild to moderate anti-inflammatory effect (0.68% inhibition for 100 mg/kg and 2.74% for 300 mg/kg) approximately 2 h after λ-carrageenan administration, corresponding to the early phase of carrageenan-induced acute inflammation, which is primarily associated with the release of histamine, serotonin, and other early inflammatory mediators. In contrast, a more pronounced anti-inflammatory effect (12% inhibition for 100 mg/kg and 10.67% inhibition) was observed approximately 4 h after carrageenan administration, with the maximum inhibition occurring during the later phase, when prostaglandin production and other inflammatory mediators contribute more prominently to paw edema. These findings suggest that the aqueous extract of *P. minus* may exert greater inhibitory effects on the later phase of acute inflammation, potentially through modulation of prostaglandin-mediated inflammatory pathways [18].

Experimental studies using carrageenan-induced paw edema models have demonstrated that PM extracts significantly reduce inflammation by inhibiting the synthesis of prostaglandins and leukotrienes, suggesting a dual inhibition of the cyclooxygenase (COX) and lipoxygenase (5-LOX) pathways [16].

Similar anti-inflammatory effects have also been observed in vivo in related species, such as Polygonum hydropiper [13,29]. Collectively, these findings provide evidence that PM may exert its anti-inflammatory effects through the dual inhibition of COX and 5-LOX enzymatic pathways, as demonstrated in both in vitro and in vivo experimental models [31].

**Table 2:** Pharmacological Studies on PM (anti-inflammatory, antioxidant, and analgesic activities).

References	Study Type	Extract/Compound	Model/Method	Pharmacological Activity	Key Findings
[24]	Phytochemical analysis	Foliage/Leaf extract	Chemical characterization	Antioxidant potential	Identified flavonoids such as quercetin and myricetin, compounds known for strong antioxidant and anti-inflammatory activities.
[35]	In vitro	Methanol extract Leaves	DPPH and reducing power assays	Antioxidant	Demonstrated strong free-radical scavenging activity, indicating high antioxidant capacity.
[25]	Chemical analysis	Essential oil	GC–MS analysis	Antioxidant/Anti-inflammatory potential	Identified volatile compounds such as decanal, dodecanal, geraniol and citral, which possess antioxidant and anti-inflammatory properties.
[22]	Phytochemical study	Leaf extract	Biochemical analysis	Antioxidant/digestive support	Reported the presence of bioactive compounds including oxalic acid, suggesting potential medicinal properties.
[25]	Molecular/phytochemical	Plant extract	Genetic and phytochemical evaluation	Pharmacological potential	Confirmed phytochemical constituents that may contribute to anti-inflammatory and antioxidant effects.
[32]	In vitro	Young Leaves and Leaf Buds Highest accumulation of flavonoids (quercetin-3-glucuronide and rutin) (oral)	Mast cell models	Anti-inflammatory	Demonstrated inhibition of histamine release, suggesting anti-inflammatory activity through suppression of mast cell activation.
[13]	In vitro/in vivo (related species)	Young Leaves and Leaf Buds Highest accumulation of flavonoids (quercetin-3-glucuronide and rutin) (oral)	Inflammatory gene expression analysis	Anti-inflammatory	Suppressed expression of COX-2, TNF-α and NF-κB, indicating inhibition of inflammatory signaling pathways.
Recent experimental studies	In vivo	Aqueous extract of PM (Glandular Trichomes of leaves) Polygodial (oral).	Carrageenan-induced rat paw edema	Anti-inflammatory/Analgesic	Significantly reduced paw edema and inflammatory mediators, suggesting inhibition of COX and 5-LOX pathways.
Recent antioxidant studies	In vitro/in vivo	Polyphenol-rich extract	Oxidative stress markers	Antioxidant	Reduced oxidative stress markers and enhanced endogenous antioxidant enzymes such as SOD and GPx.

In vivo studies have demonstrated anti-inflammatory and analgesic activities of *Polygonum minus* extracts. Oral administration of an aqueous *P. minus* extract at doses of 100 and 300 mg/kg significantly reduced carrageenan-induced paw edema in rats, with the anti-inflammatory effect being most evident at 4 h after carrageenan administration. In addition, aqueous and methanolic leaf extracts of *P. minus* have demonstrated analgesic activity in experimental models, including the acetic acid-induced writhing test and the formalin test. At the molecular level, the sesquiterpene polygodial has been shown to interact with transient receptor potential channels, including TRPV1, suggesting a potential role for sensory ion-channel modulation in its pharmacological actions. However, the specific contribution of polygodial to the analgesic activity of *P. minus* remains to be established [13,18].

#### 3.2.2. Antioxidant Activity

Oxidative stress plays a major role in the development and progression of inflammatory diseases. PM is recognized as a rich source of natural antioxidants, largely because of its high content of phenolic compounds and flavonoids.

Studies have reported that PM extracts possess substantial antioxidant and free-radical scavenging activities, as evidenced by their ability to scavenge DPPH, ABTS, superoxide, and nitric oxide radicals and to exhibit ferric-reducing antioxidant activity. In cellular models PM exhibit strong free-radical scavenging activity, which helps neutralize reactive oxygen species (ROS) and protect cellular components from oxidative damage. These antioxidant effects may contribute indirectly to its anti-inflammatory and protective biological activities [36].

In cell-free antioxidant assays, methanolic leaf extracts exhibit a DPPH radical scavenging IC_50_ ranging between 8.6 μg/mL and 15.2 μg/mL, demonstrating comparable activity to standard BHT IC_50_ = 18.1 μg/mL and Trolox IC_50_ = 4.8 μg/mL. In LPS-stimulated RAW 264.7 macrophage models, *P. minus* leaf fractions inhibit nitric oxide (NO) production with an IC_50_ of 22.4–45.1 μg/mL alongside direct enzymatic inhibition of recombinant COX-2 IC_50_ = 18.3 μg/mL [13,18,37].

Isolated polygodial appears to have limited direct free-radical scavenging activity compared with conventional phenolic antioxidants such as BHT (butylated hydroxytoluene) and Trolox, standard reference compounds widely used in antioxidant assays. Unlike BHT, which directly neutralizes free radicals and lipid peroxides through hydrogen donation from its phenolic hydroxyl group, polygodial lacks a phenolic hydroxyl group and is therefore not expected to function as a classical chain-breaking antioxidant. The antioxidant capacity observed in whole *Polygonum minus* extracts is more likely to be associated with their polyphenolic and flavonoid constituents, including quercetin, rutin, and myricetin.

Rather than acting primarily through direct radical scavenging, polygodial may exert indirect antioxidant or cytoprotective effects via its highly reactive α,β-unsaturated dialdehyde group, which can interact with cellular thiols and influence redox-sensitive signaling pathways. Therefore, polygodial and BHT represent different modes of antioxidant action: BHT acts mainly as a direct chemical radical scavenger, whereas polygodial may modulate cellular stress-response mechanisms. Consequently, the antioxidant activity of *P. minus* should be considered a combined effect of multiple constituents, rather than being attributed primarily to polygodial [38,39,40].

#### 3.2.3. Analgesic Activity

In addition to its anti-inflammatory properties, PM has demonstrated promising analgesic activity in various experimental models, primarily through its ability to modulate inflammatory and nociceptive pathways (Table 2). Pain associated with inflammation is typically mediated by the release of inflammatory mediators such as prostaglandins, bradykinin, and pro-inflammatory cytokines, which sensitize peripheral nociceptors and enhance pain perception. Bioactive compounds present in PM, particularly flavonoids such as quercetin and myricetin, are known to exert analgesic effects by inhibiting the synthesis and release of these mediators, also reducing nociceptive signaling and pain perception [18].

Experimental studies have shown that PM extracts significantly reduce pain responses in chemical-induced nociception models, such as the acetic acid-induced writhing test and the formalin test. The reduction in writhing responses suggests a peripheral analgesic effect, likely mediated through the inhibition of prostaglandin synthesis. In the formalin test, PM has been reported to attenuate both the early neurogenic phase and the late inflammatory phase of pain, indicating that it may exert both central and peripheral analgesic actions [31].

Peripheral antinociceptive evaluations using acetic acid-induced abdominal writhing in mice revealed an ED_50_ of 120–180 mg/kg b.w. p.o., effectively reducing visceral pain behavior to a degree comparable to aspirin (100 mg/kg p.o.). At the central nociceptive level, isolated bioactives such as polygodial exhibit an intraperitoneal (i.p.) ED_50_ of 15.4 mg/kg in thermal hot plate and tail-flick tests. This dual systemic action highlights the capacity of *P. minus* to attenuate both peripheral inflammatory cascades and central pain transmission [13,18].

Mechanistically, the analgesic activity of PM is associated with the inhibition of key inflammatory pathways, including cyclooxygenase (COX) and 5-lipoxygenase (5-LOX), which are involved in the metabolism of arachidonic acid, leading to reduced production of prostaglandins and leukotrienes. Unlike many conventional non-steroidal anti-inflammatory drugs (NSAIDs), which primarily inhibit COX-mediated prostaglandin synthesis, PM extracts appear to inhibit both COX and 5-LOX enzymes. This dual inhibitory mechanism may help reduce the production of inflammatory mediators such as prostaglandins (PGs) and leukotrienes (LTs), thereby preventing the accumulation of inflammatory factors that contribute to tissue damage [41].

Although direct analgesic studies on PM remain limited, supporting evidence can be drawn from in vivo and in vitro pharmacological investigations. In carrageenan-induced paw edema models, administration of aqueous extracts of PM at doses of 100 and 300 mg/kg body weight significantly reduced inflammation at 4 h, with percentage inhibition of approximately 12.0% and 10.67%, respectively (*p* < 0.01) compared with control. These findings indicate suppression of prostaglandin-mediated inflammatory processes, which are closely linked to peripheral pain mechanisms. In vitro studies demonstrated that 100 µg/mL ethanolic extracts of PM produced 100% inhibition of cyclooxygenase-1 (COX-1) with complete inhibition of 5-lipoxygenase (5-LOX) activity. It also showed moderate inhibition of COX-2 (approximately 25%), suggesting a dual inhibitory effect on arachidonic acid metabolism pathway [16].

Preclinical studies provide direct evidence supporting the analgesic potential of *Polygonum minus*. Christapher et al. demonstrated that both aqueous and methanolic leaf extracts of *P. minus* produced significant analgesic effects in experimental animals using acetic acid-induced writhing, tail-immersion, and formalin-induced nociception models. Extracts were administered at 100 and 200 mg/kg. Generally, the methanolic extract produced better analgesic activity compared to the aqueous extract, although the effects were not consistently equivalent to the standard analgesic treatment. The activity observed across both chemical and thermal nociception models suggests that *P. minus* may modulate both peripheral and central nociceptive mechanisms. These findings provide direct preclinical support for the traditional use of *P. minus* further investigation of its bioactive constituents and analgesic mechanisms [13].

During inflammation, excessive production of reactive oxygen species (ROS) contributes to the sensitization of peripheral nociceptors and enhances pain transmission by activating intracellular signaling pathways such as nuclear factor-kappa B (NF-κB) and mitogen-activated protein kinase (MAPK). These pathways promote the expression of pro-inflammatory mediators, including cyclooxygenase-2 (COX-2), inducible nitric oxide synthase (iNOS) and cytokines, which further amplify nociceptive signaling.

Bioactive compounds, particularly flavonoids such as quercetin and myricetin, as well as phenolic constituents and essential oils found in PM, exhibit strong free-radical scavenging activity. These compounds help neutralize ROS, thereby reducing oxidative stress and preventing the activation of redox-sensitive inflammatory pathways [29]. In addition, it limits the oxidative stress–induced sensitization of nociceptive neurons. By limiting ROS-mediated neuronal sensitization, PM may reduce hyperalgesia and attenuate pain perception. In addition, its antioxidant activity contributes to the preservation of endogenous antioxidant defense systems, including SOD and GPx, further protecting tissues from oxidative damage [18].

The combined inhibition of oxidative stress and inflammatory mediators suggests that the analgesic effects of PM are not solely dependent on the suppression of prostaglandin synthesis via cyclooxygenase pathways but also involve modulation of ROS-driven signaling mechanisms. This dual action highlights the therapeutic potential of PM as a natural analgesic agent, particularly in conditions where oxidative stress and inflammation are closely intertwined. Collectively, these findings support the potential of PM as a natural analgesic agent, particularly in the management of inflammatory pain [18].

**Figure 2:**
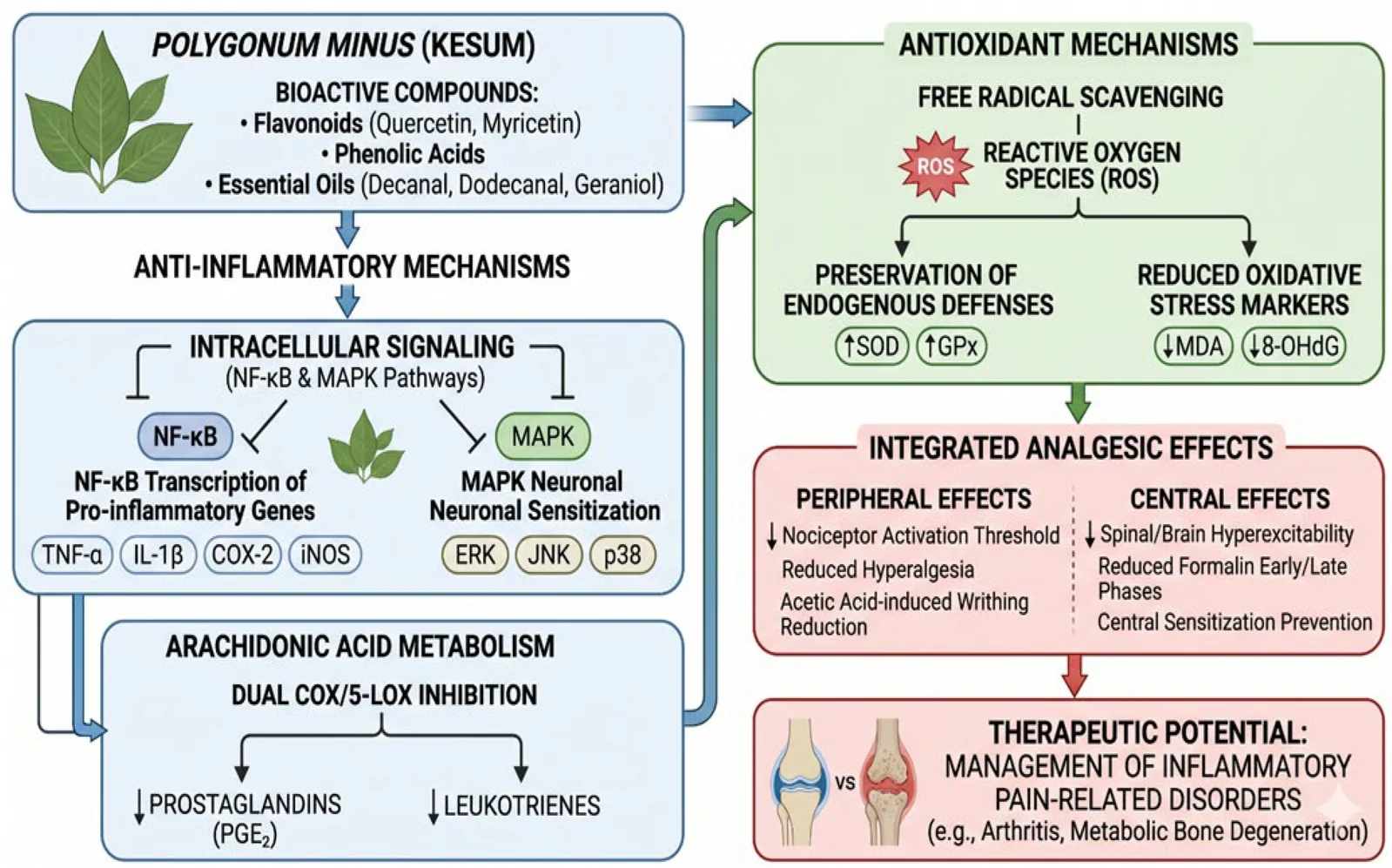
Mechanisms of action of *Polygonum minus* (Kesum).

Therefore, the investigation of the anti-inflammatory and analgesic properties of PM is of considerable scientific interest. Understanding the underlying mechanisms may contribute to the development of novel natural therapeutic agents derived from natural products for the management of inflammatory pain-related disorders [42] *(*Figure 2).

#### 3.2.4. Organ-Level Therapeutic Potential of Polygonum minus

The antioxidant and anti-inflammatory properties of *Polygonum minus* (PM) may extend beyond the musculoskeletal system and influence the function and integrity of several organs. In the liver, its antioxidant activity may reduce ROS-mediated oxidative injury and lipid peroxidation, while suppression of inflammatory signaling may help attenuate hepatic inflammation. In the gastrointestinal tract, PM has demonstrated gastroprotective and antiulcer effects in experimental models, which may be associated with its ability to reduce oxidative stress and inflammatory responses. In the brain, the antioxidant properties of PM may help protect neuronal tissue from oxidative damage, while modulation of neuroinflammatory pathways may contribute to its reported effects on cognitive function. In the cardiovascular system, attenuation of oxidative stress and inflammatory signaling may potentially protect vascular tissues from endothelial dysfunction and inflammation. PM has also demonstrated antimicrobial activity, which may complement its anti-inflammatory effects during infection-associated inflammatory responses. Collectively, these findings suggest that the antioxidant and anti-inflammatory activities of PM may have broader organ-protective effects; however, most of the available evidence remains preclinical, and further studies are required to establish the specific mechanisms, effective doses, safety, and clinical relevance of these effects. In the central nervous system, *P. minus* modulates the peripheral–central neuroinflammatory axis and cholinergic signaling. In rodent models of scopolamine-induced cognitive impairment, *P. minus* extract improved spatial memory in the Barnes maze by downregulating cortical and hippocampal acetylcholinesterase (AChE) and reducing MDA-mediated lipid peroxidation [29]. Clinically, a randomized double-blind trial confirmed these pro-cognitive effects, showing significant enhancements in executive function, working memory, and reaction time, alongside reduced stress and mood disturbances [43].

In cancer cell lines, such as HepG2 cells, *P. minus* extracts have been reported to induce apoptosis via the mitochondrial pathway. This effect is associated with an increase in the expression of pro-apoptotic proteins, particularly Bax, together with downregulation of the anti-apoptotic protein Bcl-2. These changes promote disruption of mitochondrial membrane integrity and loss of mitochondrial membrane potential (ΔΨm), which facilitates the release of apoptogenic factors and subsequently activates the intrinsic apoptotic cascade [44].

In non-cancerous and neuronal tissues, *P. minus* may protect mitochondrial integrity by reducing oxidative stress and scavenging reactive oxygen species (ROS) in the mitochondrial environment. Its antioxidant activity may enhance endogenous mitochondrial defense mechanisms by increasing the activity or expression of antioxidant enzymes, particularly superoxide dismutase (SOD) and glutathione peroxidase (GPx). These enzymes help neutralize ROS and limit ROS-induced lipid peroxidation, thereby reducing the accumulation of malondialdehyde (MDA) in mitochondrial membranes. Collectively, these effects may help preserve mitochondrial membrane integrity and maintain normal mitochondrial function [28].

#### 3.2.5. Potential Interaction of *Polygonum minus* with TrkA-Related Signaling

There is currently no empirical evidence demonstrating that *Polygonum minus* extracts or its bioactive constituents, such as polygodial, quercetin, or rutin, directly bind to or act as agonists or antagonists at the tropomyosin receptor kinase A (TrkA) receptor. Instead, its potential neurotrophic effects may involve indirect modulation of neurotrophic signaling; for example, long-term administration of *P. minus* has been reported to increase central brain-derived neurotrophic factor (BDNF) expression [45]. BDNF primarily signals through TrkB rather than TrkA, the latter being predominantly activated by nerve growth factor (NGF). In addition, the sensory and antinociceptive effects attributed to certain *P. minus* constituents, particularly polygodial, appear to involve transient receptor potential (TRP) channels, including TRPA1 and TRPV1, rather than direct interaction with neurotrophic tyrosine kinase receptors such as TrkA [42].

#### 3.2.6. Phytochemical Diversity of *Polygonum minus*

Although several bioactive compounds identified in *P. minus* are also present in other medicinal plants, the plant is characterized by a diverse phytochemical profile comprising flavonoids, drimane-type sesquiterpenes, aliphatic aldehydes, and other mono- and sesquiterpenoids. Quercetin, myricetin, and rutin represent important polyphenolic constituents, whereas decanal and dodecanal are prominent components of its volatile fraction. In addition, polygodial is a structurally distinct drimane sesquiterpene dialdehyde with potential relevance to sensory nociceptive signaling. These compounds differ substantially in their chemical structures and molecular targets, suggesting that the biological effects of *P. minus* are unlikely to arise from a single pharmacophore. Rather, the distinctive combination of chemically diverse constituents may produce complementary effects on oxidative stress, inflammatory signaling, and nociceptive pathways. Thus, the potential uniqueness of *P. minus* lies primarily in its characteristic phytochemical composition and the convergence of multiple bioactive pathways, rather than in the presence of a single species-specific compound [13,37].

#### 3.2.7. Toxicity and Safety of *Polygonum minus*

Toxicological evaluation is essential to establish the safety and appropriate dosage range of plant-derived products. Available preclinical evidence suggests that *Polygonum minus* has a relatively favorable safety profile. An acute and 28-day oral toxicity study of an aqueous *P. minus* extract in Wistar rats reported no mortality, behavioral abnormalities, significant changes in food intake, organ weights, hematological parameters, or evidence of hepatic, renal, or neurological toxicity, with a reported NOAEL of 1000 mg/kg body weight [46]. Similarly, acute administration of methanolic *P. minus* extract at up to 2000 mg/kg in female Sprague–Dawley rats produced no apparent behavioral, physiological, or gross pathological abnormalities. Subchronic administration at doses of 250–2000 mg/kg also produced no significant alterations in body weight, food and water intake, behavior, hematological or biochemical parameters, or major organ weights [47].

The limited human evidence is also encouraging. A randomized, double-blind, placebo-controlled trial involving healthy men reported no clinically significant changes in hepatic or renal function following 12 weeks of supplementation with an aqueous *P. minus* extract at 100 mg/day, based on measurements of AST, ALT, alkaline phosphatase, bilirubin, BUN, creatinine, and eGFR [43]. Collectively, these findings suggest that *P. minus* extracts are well tolerated at the doses investigated. However, differences in extraction methods, doses, duration of exposure, and study populations should be considered, and further well-controlled clinical studies are required to establish long-term safety and define the therapeutic dose range in humans.

## 4. Future Therapeutic Potential and Research Gaps

Because oxidative stress and chronic endothelial inflammation are core drivers of cardiovascular pathologies (atherosclerosis, hypertension, ischemic injury), the dual antioxidant–anti-inflammatory mechanisms of *P. minus* underscore its strong translational potential as a therapeutic functional food or botanical nutraceutical [29]. Although numerous studies have demonstrated the anti-inflammatory, antioxidant, and analgesic activities of PM, several areas require further investigation. Most current studies are limited to in vitro experiments and animal models, and there is a lack of clinical studies evaluating its therapeutic efficacy in humans.

Future research should focus on the identification and isolation of the bioactive compounds responsible for the observed pharmacological effects, as well as on elucidating their underlying molecular mechanisms of action. In addition, comprehensive evaluation of dose–response relationships and toxicity profiles are essential to establish efficacy, safety, and therapeutic potential, thereby strengthening the overall scientific validity of the study.

## 5. Conclusions

Although antioxidant, anti-inflammatory, and analgesic activities are common among medicinal plants, *Polygonum minus* possesses a distinctive combination of bioactive constituents that may contribute to its multi-target pharmacological profile. In addition to flavonoids such as quercetin and myricetin and its aldehyde-rich essential oil, *P. minus* has been reported to contain polygodial, a bioactive sesquiterpene dialdehyde that has attracted particular interest because of its effects on sensory transient receptor potential (TRP) channels, including TRPA1 and TRPV1. These mechanisms are relevant to nociception and may complement the antioxidant and anti-inflammatory actions associated with the plant’s flavonoids and volatile constituents. Thus, the potential distinctiveness of *P. minus* lies not in a single exclusive compound, but in the co-occurrence of polygodial, flavonoids, and characteristic volatile aldehydes within a traditionally important Southeast Asian medicinal and culinary plant, together with evidence suggesting activity across oxidative, inflammatory, and sensory nociceptive pathways. Given its broad spectrum of biological activities and favorable safety profile, PM represents a potential natural therapeutic agent for the management of inflammatory pain-associated conditions, including arthritis, metabolic bone disorders, and other chronic inflammatory diseases.

## Figures and Tables

**Table 1:** Major phytochemical constituents of PM and their reported biological activities.

Compound/Class	Type of Compound	Reported Biological Activities	References
Myricetin	Flavonoid (Flavanol)	Antioxidant, anti-inflammatory, free-radical scavenging, inhibition of pro-inflammatory cytokines	[23,24]
Quercetin	Flavonoid (Flavanol)	Anti-inflammatory, antihistamine activity, inhibition of mast cell degranulation, antioxidant	[24,32]
6,7-4′,5′-Dimethylenedioxy-3,5,3′-trimethoxyflavone	Methylated flavonoid	Anti-inflammatory and antioxidant potential	[23]
6,7-Methylenedioxy-5,3′,4′,5′-tetramethoxyflavone	Flavone derivative	Antioxidant and anti-inflammatory activity	[23]
Decanal	Aliphatic aldehyde (essential oil)	Antimicrobial, aromatic compound contributing to flavor	[25]
Dodecanal	Aliphatic aldehyde (essential oil)	Antimicrobial, antioxidant potential	[25]
Geraniol	Monoterpenoid alcohol	Antioxidant, antimicrobial, anti-inflammatory	[26]
Geranial (Citral)	Monoterpenoid aldehyde	Anti-inflammatory, antimicrobial, anticancer activity	[26]
β-Caryophyllene	Sesquiterpene	Anti-inflammatory, analgesic, immunomodulatory	[25]
Oxalic acid	Organic acid	May contribute to digestive enhancement activity	[22]
Polygonumins	Steroid glycoside	Cytotoxic activity against selected cancer cell lines	[33]
Polygodial	drimane-type sesquiterpene dialdehyde	Potent TRPV1 agonist causing peripheral analgesia	[33]

## Data Availability

No new data were created or analyzed in this study. Data sharing is not applicable to this article.

## Abbreviations

The following abbreviations are used in this manuscript:

PM	*Polygonum minus*
COX	cyclooxygenase
5-LOX	lipoxygenase
NF-κB	nuclear factor-kappa B
TNF-α	tumor necrosis factor-α
IL-1	interleukin-1
IL-6	interleukin-6
IL-8	interleukin-8
IL-12	interleukin-12
MAPK	mitogen-activated protein kinase
JAK-STAT	Janus kinase–signal transducer and activator of transcription
iNOS	inducible nitric oxide synthase
ROS	reactive oxygen species
DNA	DNA
PRRs	pattern recognition receptors
PAMPs	pathogen-associated molecular patterns
DAMPs	damage-associated molecular patterns
TLRs	Toll-like receptors
CRP	C-reactive protein
SOD	superoxide dismutase
GPx	glutathione peroxidase
NOX	NADPH oxidase
MDA	malondialdehyde
8-OHdG	8-hydroxy-2′-deoxyguanosine
IASP	International Association for the Study of Pain
ERK	extracellular signal-regulated kinase (ERK),
JNK	-Jun N-terminal kinase
PGE2	prostaglandin E2
NSAIDs	non-steroidal anti-inflammatory drugs
LTs	leukotrienes
PGs	prostaglandins

## References

[1] Medzhitov R. (2008). Origin and Physiological Roles of Inflammation. Nature.

[2] Medzhitov R. (2010). Inflammation 2010: New Adventures of an Old Flame. Cell.

[3] Chen L., Deng H., Cui H., Fang J., Zuo Z., Deng J., Li Y., Wang X., Zhao L. (2017). Inflammatory Responses and Inflammation-Associated Diseases in Organs. Oncotarget.

[4] Serhan C.N., Savill J. (2005). Resolution of Inflammation: The Beginning Programs the End. Nat. Immunol..

[5] Kumar V., Aster J.C., Abul K. (2015). Abbas Robbins and Cotran Pathologic Basis of Disease.

[6] Chatterjee S. (2016). Oxidative Stress, Inflammation, and Disease. Oxidative Stress and Biomaterials.

[7] Furman D., Campisi J., Verdin E., Carrera-Bastos P., Targ S., Franceschi C., Ferrucci L., Gilroy D.W., Fasano A., Miller G.W. (2019). Chronic Inflammation in the Etiology of Disease across the Life Span. Nat. Med..

[8] Punchard N.A., Whelan C.J., Adcock I. (2004). The Journal of Inflammation. J. Inflamm..

[9] Kumar V., Abbas A.K., Aster J.C. (2020). Patholgic Basis of Disease.

[10] Luo X., Xie D., Hu J., Su J., Xue Z. (2022). Oxidative Stress and Inflammatory Biomarkers for Populations with Occupational Exposure to Nanomaterials: A Systematic Review and Meta-Analysis. Antioxidants.

[11] Nathan C. (2002). Points of Control in Inflammation. Nature.

[12] Rittirsch D., Flierl M.A., Ward P.A. (2008). Harmful Molecular Mechanisms in Sepsis. Nat. Rev. Immunol..

[13] Christapher P., Parasuraman S., Christina J., Asmawi M.Z., Vikneswaran M. (2015). Review on *Polygonum minus*. Huds, a Commonly Used Food Additive in Southeast Asia. Pharmacogn. Res..

[14] Baharum S.N., Bunawan H., Ghani M.A., Mustapha W.A.W., Noor N.M. (2010). Analysis of the Chemical Composition of the Essential Oil of *Polygonum minus* Huds. Using Two-Dimensional Gas Chromatography-Time-of-Flight Mass Spectrometry (GC-TOF MS). Molecules.

[15] Bunawan H., Choong C.Y., Md-Zain B.M., Baharum S.N., Noor N.M. (2011). Molecular Systematics of *Polygonum minus* Huds. Based on ITS Sequences. Int. J. Mol. Sci..

[16] Al-Khayri J.M., Sahana G.R., Nagella P., Joseph B.V., Alessa F.M., Al-Mssallem M.Q. (2022). Flavonoids as Potential Anti-Inflammatory Molecules: A Review. Molecules.

[17] Deuis J.R., Dvorakova L.S., Vetter I. (2017). Methods Used to Evaluate Pain Behaviors in Rodents. Front. Mol. Neurosci..

[18] George A., Chinnappan S., Chintamaneni M., Kotak C.V., Choudhary Y., Kueper T., Radhakrishnan A.K. (2014). Anti-Inflammatory Effects of *Polygonum minus* (Huds) Extract (Lineminus^TM^) in In-Vitro Enzyme Assays and Carrageenan Induced Paw Edema. BMC Complement. Altern. Med..

[19] Yang Y., Yu T., Jang H.-J., Byeon S.E., Song S.-Y., Lee B.-H., Rhee M.H., Kim T.W., Lee J., Hong S. (2012). In Vitro and In Vivo Anti-Inflammatory Activities of Polygonum Hydropiper Methanol Extract. J. Ethnopharmacol..

[20] Woolf C.J. (2010). What Is This Thing Called Pain?. J. Clin. Investig..

[21] Tappe-Theodor A., King T., Morgan M.M. (2019). Pros and Cons of Clinically Relevant Methods to Assess Pain in Rodents. Neurosci. Biobehav. Rev..

[22] Gregory N.S., Harris A.L., Robinson C.R., Dougherty P.M., Fuchs P.N., Sluka K.A. (2013). An Overview of Animal Models of Pain: Disease Models and Outcome Measures. J. Pain.

[23] Martinov T., Mack M., Sykes A., Chatterjea D. (2013). Measuring Changes in Tactile Sensitivity in the Hind Paw of Mice Using an Electronic von Frey Apparatus. J. Vis. Exp..

[24] Basbaum A.I., Bautista D.M., Scherrer G., Julius D. (2009). Cellular and Molecular Mechanisms of Pain. Cell.

[25] Lu R., Schmidtko A. (2013). Direct Intrathecal Drug Delivery in Mice for Detecting In Vivo Effects of CGMP on Pain Processing. Methods Mol. Biol..

[26] Jang Y., Kim M., Hwang S.W. (2020). Molecular Mechanisms Underlying the Actions of Arachidonic Acid-Derived Prostaglandins on Peripheral Nociception. J. Neuroinflamm..

[27] Yaacob K.B. (1990). Essential Oil of *Polygonum minus* Huds. J. Essent. Oil Res..

[28] George A., Ng C.P., O’Callaghan M., Jensen G.S., Wong H.J. (2014). In Vitro and Ex-Vivo Cellular Antioxidant Protection and Cognitive Enhancing Effects of an Extract of *Polygonum minus* Huds (Lineminus^TM^) Demonstrated in a Barnes Maze Animal Model for Memory and Learning. BMC Complement. Altern. Med..

[29] Hamid A.A., Aminuddin A., Yunus M.H.M., Murthy J.K., Hui C.K., Ugusman A. (2020). Antioxidative and Anti-Inflammatory Activities of *Polygonum minus*: A Review of Literature. Rev. Cardiovasc. Med..

[30] Mendes G.L., Santos A.R.S., Campos M.M., Tratsk K.S., Yunes R.A., Filho V.C., Calixto J.B. (1998). Anti-Hyperalgesic Properties of the Extract and of the Main Sesquiterpene Polygodial Isolated from the Barks of Drymis Winteri (Winteraceae). Life Sci..

[31] Mendes G.L., Santos A.R.S., Malheiros A., Filho V.C., Yunes R.A., Calixto J.B. (2000). Assessment of Mechanisms Involved in Antinociception Caused by Sesquiterpene Polygodial. J. Pharmacol. Exp. Ther..

[32] Imelda F., Faridah D.N., Kusumaningrum H.D. (2014). Bacterial Inhibition and Cell Leakage by Extract of *Polygonum minus* Huds. Leaves. Int. Food Res. J..

[33] Maarof N.D., Ali Z.M., Nor N.M., Hassan M. (2010). Isolation of NADP+-Geraniol Dehydrogenase from *Polygonum minus*. National biotechnology Seminar, Kannur, Kerala, India.

[34] Vimala S., Rohana S., Rashih A.A., Juliza M. (2012). Antioxidant Evaluation in Malaysian Medicinal Plant: Persicaria Minor (Huds.) Leaf. Sci. J. Med. Clin. Trials.

[35] Aggarwal D., Chaudhary M., Mandotra S.K., Tuli H.S., Chauhan R., Joshi N.C., Kaur D., Dufossé L., Chauhan A. (2025). Anti-Inflammatory Potential of Quercetin: From Chemistry and Mechanistic Insight to Nanoformulations. Curr. Res. Pharmacol. Drug Discov..

[36] Abdullah M.Z., Mohd Ali J., Abolmaesoomi M., Abdul-Rahman P.S., Hashim O.H. (2017). Anti-Proliferative, In Vitro Antioxidant, and Cellular Antioxidant Activities of the Leaf Extracts from *Polygonum minus* Huds: Effects of Solvent Polarity. Int. J. Food Prop..

[37] Qader S.W., Abdulla M.A., Chua L.S., Najim N., Zain M.M., Hamdan S. (2011). Antioxidant, Total Phenolic Content and Cytotoxicity Evaluation of Selected Malaysian Plants. Molecules.

[38] Yehye W.A., Rahman N.A., Ariffin A., Abd Hamid S.B., Alhadi A.A., Kadir F.A., Yaeghoobi M. (2015). Understanding the Chemistry behind the Antioxidant Activities of Butylated Hydroxytoluene (BHT): A Review. Eur. J. Med. Chem..

[39] Christapher P., Xin T., Kiun C., Leng L., Fu N., Yuan G., Parasuraman S., Vikneswaran M. (2015). Evaluation of Analgesic, Anti-Inflammatory, Antipyretic and Antiulcer Effect of Aqueous and Methanol Extracts of Leaves of *Polygonum minus* Huds. (Polygonaceae) in Rodents. Arch. Med. Health Sci..

[40] Murad M., Aminah A., Wan Aida W.M. (2011). Total Phenolic Content and Antioxidant Activity of Kesum (*Polygonum minus*), Ginger (*Zingiber officinale)* and Turmeric (*Curcuma longa*) Extract. Int. Food Res. J..

[41] Weng Z., Zhang B., Asadi S., Sismanopoulos N., Butcher A., Fu X., Katsarou-Katsari A., Antoniou C., Theoharides T.C. (2012). Quercetin Is More Effective than Cromolyn in Blocking Human Mast Cell Cytokine Release and Inhibits Contact Dermatitis and Photosensitivity in Humans. PLoS ONE.

[42] Escalera J., von Hehn C.A., Bessac B.F., Sivula M., Jordt S.-E. (2008). TRPA1 Mediates the Noxious Effects of Natural Sesquiterpene Deterrents. J. Biol. Chem..

[43] Udani J.K. (2013). Effects of SuperUlam on Supporting Concentration and Mood: A Randomized, Double-Blind, Placebo-Controlled Crossover Study. Evid.-Based Complement. Altern. Med..

[44] Mohd Ghazali M.A., Al-Naqeb G., Krishnan Selvarajan K., Hazizul Hasan M., Adam A. (2014). Apoptosis Induction by *Polygonum minus* Is Related to Antioxidant Capacity, Alterations in Expression of Apoptotic-Related Genes, and S-Phase Cell Cycle Arrest in HepG2 Cell Line. Biomed Res. Int..

[45] Bashir M.I., Abdul Aziz N.H.K., Noor D.A.M. (2022). Antidepressant-like Effects of *Polygonum minus* Aqueous Extract in Chronic Ultra-Mild Stress-Induced Depressive Mice Model. Behav. Sci..

[46] Ming Y.K., Parasuraman S., Asmawi M.Z. (2013). Acute and sub-acute oral toxicity of *Polygonum minus* aqueous extract (biotropics^®^ PM101) in Wistar rats. Int. J. Pharm. Pharm. Sci..

[47] Christapher P.V., Parasuraman S., Asmawi, Mohd Z., Murugaiyah V. (2017). Acute and Subchronic Toxicity Studies of Methanol Extract of *Polygonum minus* Leaves in Sprague Dawley Rats. Regul. Toxicol. Pharmacol..

